# Artificial intelligence in triage of COVID-19 patients

**DOI:** 10.3389/frai.2024.1495074

**Published:** 2024-12-18

**Authors:** Yuri Oliveira, Iêda Rios, Paula Araújo, Alinne Macambira, Marcos Guimarães, Lúcia Sales, Marcos Rosa Júnior, André Nicola, Mauro Nakayama, Hermeto Paschoalick, Francisco Nascimento, Carlos Castillo-Salgado, Vania Moraes Ferreira, Hervaldo Carvalho

**Affiliations:** ^1^School of Medicine, University of Brasilia, Brasilia, Brazil; ^2^School of Health Sciences, University of Brasilia, Brasilia, Brazil; ^3^University Hospital of Brasilia, University of Brasilia, Brasilia, Brazil; ^4^Hospital of Tropical Diseases, Federal University of Tocantins, Araguaína, Brazil; ^5^University Hospital, Federal University of Vale do São Francisco, Petrolina, Brazil; ^6^Institute of Health Sciences, Federal University of Pará, Belém, Brazil; ^7^University Hospital Cassiano Antônio de Moraes, Federal University of Espírito Santo, Vitória, Brazil; ^8^University Hospital, Federal University of Grande Dourados, Dourados, Brazil; ^9^Department of Electrical Engineering, University of Brasilia, Brasilia, Brazil; ^10^School of Public Health, Johns Hopkins University, Baltimore, MD, United States

**Keywords:** artificial intelligence, machine learning, clinical data, COVID-19, outcome prediction, prediction algorithms, triage

## Abstract

In 2019, COVID-19 began one of the greatest public health challenges in history, reaching pandemic status the following year. Systems capable of predicting individuals at higher risk of progressing to severe forms of the disease could optimize the allocation and direction of resources. In this work, we evaluated the performance of different Machine Learning algorithms when predicting clinical outcomes of patients hospitalized with COVID-19, using clinical data from hospital admission alone. This data was collected during a prospective, multicenter cohort that followed patients with respiratory syndrome during the pandemic. We aimed to predict which patients would present mild cases of COVID-19 and which would develop severe cases. Severe cases were defined as those requiring access to the Intensive Care Unit, endotracheal intubation, or even progressing to death. The system achieved an accuracy of 80%, with Area Under Receiver Operating Characteristic Curve (AUC) of 91%, Positive Predictive Value of 87% and Negative Predictive Value of 82%. Considering that only data from hospital admission was used, and that this data came from low-cost clinical examination and laboratory testing, the low false positive rate and acceptable accuracy observed shows that it is feasible to implement prediction systems based on artificial intelligence as an effective triage method.

## 1 Introduction

Initiated in 2019, Coronavirus Disease (or COVID-19), a respiratory infection caused by Severe Acute Respiratory Syndrome Coronavirus 2 (SARS-CoV-2), rapidly spread worldwide, reaching pandemic status as early as March 2020 (Sott et al., 2022; Mohamed et al., 2020). According to the World Health Organization (WHO), by October 4, 2023, there were already 771,151,224 confirmed cases and 6,960,783 deaths globally (World Health Organization, 2023). COVID-19 is primarily characterized by pneumonia symptoms, including fever, fatigue, and dry cough. However, other symptoms such as gastrointestinal alterations, anosmia, or even ophthalmological changes can also be observed (Luo et al., 2022). Severe cases require intensive care, with the need for endotracheal intubation and mechanical ventilation, leading to death in extreme cases (Luo et al., 2022; Ou et al., 2020; Martono and Mulyanti, 2023; Fang et al., 2020).

The rapid increase in infection numbers highlighted the unpreparedness of healthcare systems, affecting even developed countries. There were shortages of qualified professionals (Buonsenso et al., 2021; Yoshioka-Maeda et al., 2020), medications (Kanji et al., 2020), equipment (Sandhu et al., 2022), and reagents for laboratory tests (Brinati et al., 2020; Arpaci et al., 2021). To address resource deficiencies, new strategies emerged, including changes in management approaches (Sott et al., 2022; Mohamed et al., 2020; Buonsenso et al., 2021; Yoshioka-Maeda et al., 2020), vaccine distribution (Mohamed et al., 2022), and the development of alternative techniques for treatments and diagnostics (Kanji et al., 2020; Brinati et al., 2020; Arpaci et al., 2021; Kim, 2022). Social impacts were also notable, including worsened economic situations, increased cases of domestic violence, and disruptions in the treatment of other diseases (Sott et al., 2022).

The global crisis and its devastating consequences, along with resource scarcity, have motivated the development of several artificial intelligence (AI) based tools. Algorithms have been implemented to assist in COVID-19 diagnosis, interpretation of imaging exams, prediction of case variations, drug and vaccine development, and prognosis prediction for infected patients (Brinati et al., 2020; Arpaci et al., 2021; Rahman et al., 2021; Yu et al., 2021; Isgut et al., 2023; Shakibfar et al., 2023; Wang et al., 2021; Napolitano et al., 2022; Ramírez-Del Real et al., 2022; Kamel et al., 2023).

Developing robust predictors of morbidity and mortality during a pandemic could aid in strategic healthcare planning, providing early indications, for example, of the need for hospitalization for a group of infected patients (Shakibfar et al., 2023). The quality of data used to construct a predictor is crucial, as incomplete, or poorly processed clinical data can introduce biases that hinder implementation (Isgut et al., 2023).

Being the first major pandemic of the digital era, an enormous amount of information has been collected (Isgut et al., 2023; Napolitano et al., 2022). With the goal of identifying risk factors most associated with COVID-19 severity, dozens of cohort studies, case–control studies, and case series have been conducted (Ou et al., 2020; Martono and Mulyanti, 2023; Fang et al., 2020). Based on this information, AI-based predictors of mortality and morbidity can be developed (Shakibfar et al., 2023).

Artificial Intelligence (AI) is a term that has been widely used in literature, but often with distinct definitions. This becomes more evident when this term is used in conjunction with Machine Learning (ML). Although there is no consensus, it is safe to define AI as a system that seeks to mimic human intelligence. ML, on the other hand, is considered a subset of AI and refers to systems capable of learning from datasets without explicit programming on how to make decisions (Kühl et al., 2022). Another frequently used concept is that of Artificial Neural Networks (ANNs), systems inspired by the functioning of the nervous system and capable of learning from presented data. ANNs can have various architectures and can be defined as a subset of ML (Wang et al., 2021; Haglin et al., 2019).

There are various ML methods, each more suitable for different tasks. Learning can be supervised, semi-supervised, or unsupervised, and tasks include classification, clustering, regression, localization, among others. In a classification task, such as predicting COVID-19 morbidity based on clinical data, several supervised algorithms can be used. These algorithms are called supervised because clinical data is presented with labels, i.e., in conjunction with the outcome for each patient (Uddin et al., 2019).

Within the family of supervised classification methods, different approaches can be taken, such as logistic regression, decision trees, support vector machines, Random Forest, artificial neural networks, among others. Each method has a different approach to data analysis, but they all work by finding parameters that minimize classification error. These parameters represent the learning acquired from data analysis (Uddin et al., 2019).

Artificial Neural Networks (ANNs) have gained popularity due to increased computer processing power and the availability of digital information. These algorithms are inspired by the functioning of the nervous system, where a network of artificial neurons can receive input data, such as laboratory test results, and deliver an output indicating the class to which the individual belongs—such as the presence or absence of a disease. This method involves successive nonlinear transformations to determine whether each unit (or artificial neuron) in the network will be activated, simulating neuronal depolarization (Grossi and Buscema, 2007; Rodvold et al., 2001; Buscema, 2002).

These artificial networks can learn input data patterns so well that they often memorize the labels of the presented data, resulting in overfitting and low generalization capacity for unseen data. Overfitting is a major challenge when developing a machine learning (ML)-based classifier because an overfitted system performs well on training data but poorly when used to classify new patients. An overfitted system can be likened to a doctor who correctly diagnoses only cases for which they have seen the answer before but struggles to recognize diseases in new patients. Various techniques are used to prevent overfitting, including controlling the neural network’s size, limiting learning iterations (early stopping), dropout regularization, and others (Grossi and Buscema, 2007; Rodvold et al., 2001; Buscema, 2002; Pansambal and Nandgaokar, 2023; Salehin and Kang, 2023).

Combining the extensive data collection carried out during the pandemic with artificial intelligence methods, several published studies aim at predicting patient outcomes for COVID-19. Fernandes et al. (2021) utilized data from 1,040 patients, incorporating laboratory, clinical, and demographic information. As in this study, only data collected at the time of hospital admission were considered; however, the data were collected from a single hospital. A total of 57 variables were used after excluding those with over 90% missing data and those with a correlation above 0.9. The implemented models demonstrated high predictive capability, with Area Under the Receiver Operating Characteristic Curve (AUC) values exceeding 0.91 in identifying various adverse outcomes.

Kwok et al. (2023) also achieved favorable results in developing a predictor for adverse outcomes in COVID-19 hospitalizations. Using retrospective data from 16 different hospitals, they trained ML models with 92 variables, achieving an AUC of 0.852 and an accuracy of 89%. Notably, they utilized the 6 variables that contributed most to the AI model to construct a new risk score.

In another retrospective study, this time including results from radiological examinations, Tenda et al. (2024) developed another predictor with an AUC of 0.815 in the dataset used to train the classifier and 0.770 in a dataset collected solely for model validation. In another study with retrospective multicenter data, Kamel et al. (2023) were able to predict progression to adverse outcomes. By comparing different subsets, they determined that hematological variables had the highest predictive capacity for outcomes, but the combination of all collected data resulted in better accuracy. Various tested algorithms showed 90% accuracy, with excellent sensitivity, specificity, and AUC.

Jimenez-Solem et al. (2021) also investigated the predictive capability of severe outcomes using ML. This study stands out for its analysis of different outcomes at various points during patient evaluation. Additionally, they performed external validation, demonstrating that these ML models can be utilized in patients with COVID-19.

Heldt et al. (2021) also achieved satisfactory results using only data collected during the initial moments of medical care. Additionally, Hao et al. (2022) analyzed similar outcomes and raised an important question regarding racial bias in the classifiers, with high false positive rates for hospitalization risk in Black patients.

We identified some research gaps in our literature review. The first observation relates to data collection methods. Most published studies relied on retrospective cohorts, which can introduce biases and confounding factors (Kamel et al., 2023). Additionally, most studies gathered data from a single healthcare center. Single-center studies may limit the generalizability of classification models (Kwok et al., 2023; Tenda et al., 2024; Hao et al., 2022). Possibly due to publication bias, the studies we reviewed did not thoroughly discuss data availability, as we noticed that a large portion of the analyzed variables were available for over 90% of study participants. These near-ideal datasets fail to reflect the reality of many healthcare systems during the health crisis, where multiple tests were not performed due to limited availability. This resource scarcity results in missing-not-at-random (MNAR) data, introducing biases and confounding factors that must be addressed (Isgut et al., 2023).

The main objective of this study is to assess the performance of ML algorithms as outcome predictors during COVID-19 hospitalization using only hospital admission data. Throughout this process, we aim to:

Evaluate the availability of data in real-world scenarios, particularly in resource-constrained environments where access to advanced diagnostic tests is limited, leading to a significant amount of missing data. To achieve this, we conducted a prospective multicenter cohort study, tracking patients admitted with suspected COVID-19 and collecting clinical, laboratory, and demographic information. This approach aimed to minimize the risk of bias and potential confounders typically associated with retrospective or single-center studies.

Discuss different methods for handling far from ideal datasets, with a great quantity of MNAR data and the potential biases they may introduce, covering the entire process from data preprocessing to AI model training.

Analyze the performance of classification algorithms on a dataset that has been preprocessed to reflect the clinical significance of variables, particularly by categorizing physical and laboratory examination results based on established reference values and grouping variables with similar medical significance to reduce sparsity.

Assess the capability of ML algorithms to triage hospitalized COVID-19 patients by predicting which individuals are likely to experience significant deterioration, such as requiring intubation, admission to the Intensive Care Unit (ICU), or facing a fatal outcome.

Assess the impact of different approaches to handling missing data, such as selecting variables with the highest completion rates while excluding subjects lacking data for all selected variables or using mean imputation.

Validate a methodology for the development of tools to assist in combating new diseases, starting from data collection, preprocessing, and experimenting with different algorithms.

The contributions of this research are multifaceted and include de following key points:

1. The study demonstrates the feasibility of using ML algorithms to predict a severe outcome of COVID-19 based on hospital admission data. Utilizing data from a prospective multicenter cohort, the study achieved 80% accuracy and 91% AUC. By using only hospital admission data, we developed systems capable of making early predictions, enabling important conclusions to be drawn at the beginning of a patient’s hospitalization. This early identification of at-risk patients can significantly enhance triage processes and improve clinical decision-making, ultimately contributing to better patient outcomes.
2. The article emphasizes the importance of data quality and preprocessing in developing AI predictors, addressing challenges posed by missing data and sparse variables, which are common in clinical studies, especially during crises in healthcare systems. The study employed mean imputation for missing data but acknowledged that other methods may yield better results, while utilizing medical knowledge-based strategies to group and reduce the dimensionality of sparse variables.
3. The research compares the performance of various ML algorithms, including support vector machines, random forests, and dense neural networks, providing a comprehensive analysis of accuracy, sensitivity, specificity, and AUC for each algorithm. Although these techniques are well-established and widely used, the study focuses on their performance on this specific dataset, which has its limitations and underwent a different preprocessing approach.
4. The study contributes to the literature on AI predictors for COVID-19 outcomes, particularly by utilizing data from prospective multicenter studies, which improves generalizability and applicability compared to single-center retrospective studies that may have limited external validity. Moreover, the prospective data ensures better quality control and reduces potential bias.
5. This study develops predictive models using a dataset that mirrors the practical constraints faced by healthcare systems, particularly during times of crisis. Unlike studies that rely on idealized datasets and often overlook challenges in access to diagnostic tests and other resources, our approach incorporates these limitations directly. This enables the creation of models that are not only predictive but also adaptable to real-world healthcare environments where resources are constrained.
6. The article underscores the necessity of integrating medical knowledge into the development and evaluation of AI systems, arguing that AI should complement rather than replace human expertise, while highlighting the importance of considering the feasibility, applicability, and clinical validity of AI predictors.
7. Finally, the study provides a replicable methodology for developing morbidity and mortality predictors that can be applied to other diseases. This framework, from data collection and preprocessing to algorithm selection and evaluation, can be adapted to different clinical scenarios and contribute to advancing AI-based decision support tools in healthcare.

## 2 Methods

**Figure 1:**
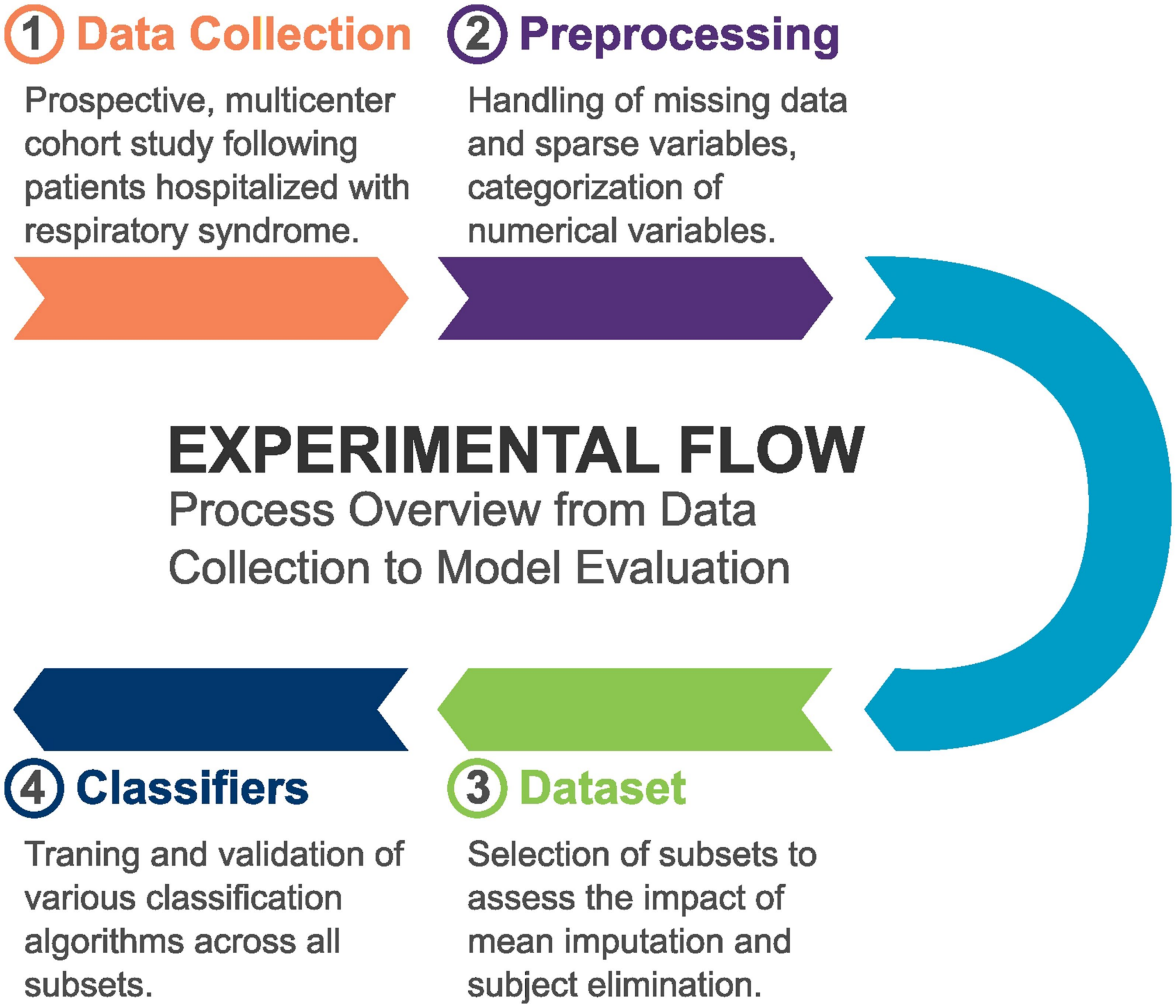
Overview of the methodology, illustrating the steps involved in data collection, preprocessing, dataset composition, and the training of machine learning classifiers using various subsets.

Our study involved several key steps in the methodology. Initially, we collected data from patients hospitalized with respiratory syndrome. Following data collection, we performed extensive preprocessing, which included cleaning the data and handling missing values. Subsequently, we composed a primary dataset along with various subsets tailored for specific analyses. These subsets were utilized to train different machine learning classifiers, enabling us to assess their performance and effectiveness in predicting outcomes related to the condition under investigation. Figure 1 illustrates this process.

### 2.1 Data collection

We conducted a prospective, multicenter, concurrent cohort study that included all patients over 18 years of age admitted for respiratory syndrome, with confirmed or suspected COVID-19, at one of the participating hospitals: University Hospital of Brasília (HUB), Brasília-DF, Brazil; Regional Hospital of Asa Norte (HRAN), Brasília-DF, Brazil; Hospital das Clínicas of the Federal University of Minas Gerais (UFMG), Belo Horizonte-MG, Brazil; Cassiano Antônio Moraes University Hospital (HUCAM), Vitória-ES, Brazil; João de Barros Barreto University Hospital of the Federal University of Pará (UFPA), Belém-PA, Brazil; University Hospital of the Federal University of São Francisco Valley (Univasf), Petrolina-PE, Brazil; University Hospital of the Federal University of Grande Dourados (UFGD), Dourados-MS, Brazil; and Tropical Diseases Hospital (HDT-UFT), Araquaína-TO, Brazil. The data collection period was from June 2020 to January 2021 and only pregnant patients were excluded from the study. Cases of respiratory syndrome were defined by the association of general symptoms such as dyspnea, decreased oxygen saturation, cyanosis, and/or radiographic lung changes.

Patients or their legal representatives authorized data collection through an informed consent form, where they were informed about all risks associated with the study, as well as the measures taken to ensure data privacy and protection. They were also notified that they could withdraw from the study at any time. To protect data privacy, identifying features were removed before preprocessing and training, with only an automatically assigned random identification number retained. Access to potentially identifying data was limited to a few team members who ensured its security. The study adhered to the ethical principles outlined in the Declaration of Helsinki and the Belmont Report, and it was approved by an independent ethics committee (Research Ethics Committee from the University of Brasília School of Medicine), under the Certificate of Ethical Appreciation Presentation number 31941420.4.1001.5558 and approval number 4.054.462. No additional interventions were performed beyond those necessary for the proper monitoring of hospitalized patients, such as blood tests and clinical examinations.

Clinical data were collected within the first 72 h of hospitalization, recording the initial clinical examination (including vital signs and identification of comorbidities), demographic information, imaging, and laboratory tests. These tests included Polymerase Chain Reaction (PCR) on sputum, nasal swabs, and serology for SARS-CoV-2, aiming to identify the presence of the coronavirus. After data collection, patients were divided into two groups: those with confirmed SARS-CoV-2 infection (either by PCR or serology) and patients with negative test results. All patients, whether COVID-19 positive or not, were further categorized into those who experienced severe complications and those who did not. Severe complications included patients requiring mechanical ventilation, endotracheal intubation, or ICU admission, as well as those who experienced cardiac arrest, severe sepsis, or who died.

### 2.2 Data preprocessing

For the development of severity prediction algorithms, the data underwent preprocessing. Due to resource scarcity during the pandemic, several tests could not be conducted on all patients, resulting in a high number of missing data points among individuals. Missing data or incompleteness is a challenge in various studies conducted during the pandemic (Isgut et al., 2023). Initially, variables collected in less than 50% of participants and constant variables (those with the same value for all participants) were removed. For example, the presence of jugular turgor, that wasn’t identified in any patient, was excluded.

When dealing with incomplete data, two approaches are possible (Isgut et al., 2023). The first involves excluding individuals who lack data for the algorithm’s variables of interest. The second approach is data imputation, where missing values are estimated to retain all individuals in the study. Various imputation methods exist; in this study, data were imputed using the means. For instance, the average hemoglobin value across all patients was used to fill in missing hemoglobin measurements for those who did not undergo the test. Both elimination and mean imputation approaches were employed in this work.

In addition to missing data, clinical conditions that were not observed in a significant number of subjects, sparse data, also pose challenges in developing predictors using data from clinical studies (Tipirneni and Reddy, 2022). Because these clinical variables are positive in a very small proportion of individuals, they do not provide much information to classifiers and increase complexity by adding to the data’s dimensionality. Several variables collected in our cohort, particularly prior diseases, symptoms, or physical examination findings, were positive in less than 1% of patients. Although they are sparse, these variables might be important to the prediction and eliminating them from the study might decrease system’s accuracy. To reduce data dimensionality without disregarding the importance of these alterations, we cataloged variables suitable for grouping or clustering based on medical knowledge. Thus, variables with similar clinical significance or those composing the same syndrome were combined using a logical “OR” operation, such that the grouping variable would be negative only if all the originating variables were also negative. With the operation, the union of previous Chronic Obstructive Pulmonary Disease, asthma or other chronic lung conditions resulted in a single variable of presence of pre-existing lung diseases. The same approach was applied to symptoms of Upper Respiratory Tract Infection, grouping presence of runny nose, postnasal drip, nasal congestion, sneezing, sore throat, difficulty swallowing and presence of facial sinus compression pain. Nonspecific viral infection symptoms were the union of muscle pain, dizziness, joint pain, skin lesions, diarrhea, abdominal pain, mental confusion, chills, enlarged lymph nodes, nausea, vomiting, loss of appetite and pale mucous membranes. Signs of pulmonary involvement were the union of presence of productive cough, chest pain, cyanosis, chest compression pain, respiratory effort, increased respiratory rate, irregular respiratory pattern, decreased chest expansion, snoring, wheezing, crackles, pinkish sputum, and pulmonary edema.

After this grouping, variables with low representation (less than 5% positive cases) were excluded from the analysis. Variables whose clinical meaning is related to another alteration, such as loss of taste and anosmia, were considered repeated and the ones with lower frequency of positive cases were removed. The distribution of variables for each class (or outcome) was not considered during variable selection to avoid bias in classifier analysis.

All numerical data was categorized. Age was categorized based on percentiles, using 0, 10, 25, 50, 75, 90 and 100% as separators. Thus, patients whose age falls at or below the 10th percentile will be assigned the value 0. Those whose age is above the 10th percentile, but equal or less than the 25th percentile will receive the value 1. Similarly, value 2 corresponds to ages between the 25th and 50th percentiles, and so on. This approach transforms age from years to a class ranging from 0 to 5.

The hemoglobin levels were categorized for female patients using the following intervals: greater than 0 and lower or equal to 6.5 g/dL; greater than 6.5 and lower or equal to 8 g/dL; greater than 8 and lower or equal to 10 g/dL; greater than 10 and lower or equal to 12 g/dL; greater than 12 and lower or equal to 16.5 g/dL; and above 16.5 g/dL. The same was done for male patients, but the value 12 was substituted to 13 g/dL and 16.5 to 18 g/dL. Consequently, a woman with a hemoglobin level of 8 g/dL was assigned category 1, while a man with a hemoglobin level of 13.7 g/dL was assigned category 4. This range of values was inspired by the National Health Institute (NSH) and National Cancer Institute (NCI) classifications for anemia and its severity.

Leukocytes, lymphocytes, platelets, and urea were categorized as low, normal, and high, also inspired by the reference values defined by the National Health Institute. Leukocytes were considered low when equal or lower than 4,000 cells/ mm^3^, high when higher than 11,000 cells/ mm^3^ and normal in between. For lymphocytes the lower boundary was 1 cell/mm^3^ and the higher was 4 cells/mm^3^. For platelets the values were 150,000 cells/mm^3^ and 400,000 cells/mm^3^, respectively, and for urea it was 15.02 mg/dL and 46.84 mg/dL.

Although measured upon patient admission, systolic and diastolic blood pressures were categorized using criteria inspired by the American Heart Association (AHA) classification for hypertension and hypotension. The range between 140 and 180 mmHg for systolic blood pressure was excluded (as there were only 2 cases above 180 mmHg). This classification approach was chosen solely to use the values as reference points, not for diagnosing or classifying arterial hypertension. Thus, the systolic pressure ranges were the following: greater than 0 and lower or equal to 90 mmHg; greater than 90 and lower or equal to 120 mmHg; greater than 120 and lower or equal to 130 mmHg; greater than 130 and lower or equal to 140 mmHg; and above 140 mmHg. For diastolic pressure, the ranges were: greater than 0 and lower or equal to 60 mmHg; greater than 60 and lower or equal to 80 mmHg; greater than 800 and lower or equal to 90 mmHg; greater than 90 and lower or equal to 120 mmHg; and above 120 mmHg.

The heart rate was categorized using 60 bpm as the cutoff value for bradycardia and 100 bpm as the cutoff value for tachycardia. The respiratory rate was defined as normal up to 16 breaths per minute (bpm), borderline between 16 and 20 bpm, elevated between 20 and 24 bpm, and very high above 24 bpm. Finally, oxygen saturation (SpO2) values equal to or below 88% were considered extremely low, between 88 and 92% inclusive as very low, between 92 and 96% inclusive as low, and above 96% as normal.

This categorization was performed to align the numerical values across different variables. For instance, hemoglobin levels varied around the number 10, while platelet counts ranged around 200,000. The difference in dynamic range and magnitude among variables could potentially lead to poorer performance in certain algorithms. Some algorithms might assign greater importance to specific variables not due to their predictive significance but based on their absolute values. Another approach that could have been used is normalization, which scales all numerical variables between 0 and 1. However, we opted for categorization because it also aligned numerical variables (such as laboratory test results) with categorical ones (such as overall health status). For example, the general condition at admission was classified as follows: 0: good, 1: mildly compromised, 2: moderately compromised, 3: severely compromised, and 4: critical. This categorization also considers how physicians analyze clinical examination results.

### 2.3 Dataset development

To assess the impact of eliminating individuals with missing data and imputation on algorithm training, we developed a dataset by selecting the 28 most complete variables—those collected from most patients. Using this dataset, we constructed 7 different subsets. One subset included all 421 patients and all 28 variables, with missing values imputed using the mean. In the remaining subsets, no imputation was performed, resulting in data loss due to incomplete records. These 6 subsets were created by varying the number of variables included, ranging from the 23 most complete variables to a set containing all 28 variables. The 7 subsets will be named A to G. The variables and number of subjects of each subset is described below:

1. Subset A, 355 subjects. Contains 23 variables:
  - Age.
  - Hemoglobin.
  - Leukocytes count.
  - Lymphocytes count.
  - Platelets count.
  - Heart rate.
  - SpO2.
  - Absence of previous disease.
  - Systemic Arterial Hypertension.
  - Diabetes Mellitus.
  - Obesity.
  - Renal insufficiency.
  - Fever.
  - Dry cough.
  - Headache.
  - Weakness.
  - Anosmia.
  - Sex.
  - Upper Respiratory Tract Infection signs.
  - Dyspnea.
  - Signs of pulmonary involvement.
  - Presence of nonspecific viral infection symptoms.
  - Pre-existing lung diseases.
2. Subset B, 335 subjects. Contains 24 variables:
  - All 23 variables from subset A.
  - General condition at admission.
3. Subset C, 309 subjects. Contains 25 variables:
  - All 24 variables from subset B.
  - Respiratory rate at admission.
4. Subset D, 285 subjects. Contains 26 variables:
  - All 25 variables from subset C.
  - Systolic blood pressure at admission.
5. Subset E, 285 subjects. Contains 27 variables:
  - All 26 variables from subset D.
  - Diastolic blood pressure at admission.
6. Subset F, 246 subjects. Contains 28 variables:
  - All 27 variables from subset E.
  - Urea.
7. Subset G, 421 subjects. Contains 28 variables:
  - Same 28 variables from subset F, with data imputation to fill missing values.

The advantage of constructing the subsets in this manner lies in its consideration of data availability when selecting variables, thereby aiming to preserve the maximum number of individuals in the groups while minimizing the elimination of patients with missing data. By reducing the amount of missing data during classifier development, we aimed to mitigate the risk of bias introduced by MNAR data while preserving as much data as possible.

By focusing on the 28 most complete variables, we ensured that the selected variables could be collected across a wide range of patients, even during a healthcare system crisis. This approach suggests that these variables represent more readily available and easily applicable examinations, ultimately enhancing the applicability of the model in real-world clinical settings. Additionally, we aimed to evaluate the balance between maintaining more variables in the subset, which could potentially increase accuracy, and the loss of individuals due to elimination, which could reduce both accuracy and generalization.

### 2.4 Classifiers development

The data was then split into training and test groups, comprising 80 and 20% of the total patients, respectively. These subsets were used to train ML algorithms. Random selection between the groups can introduce bias in accuracy analysis; for instance, when individuals that are easier to classify end up in the test group, artificially inflating the system’s accuracy. To mitigate this impact, each algorithm underwent training through 5,000 simulations, with each simulation representing a random resampling of the groups and retraining from scratch. This validation technique, known as Monte Carlo Cross-Validation, shows more accurate result even with subsets containing over 1,000 observations (Shan, 2022). During each simulation, accuracy, sensitivity, specificity, negative predictive value, positive predictive value, F1-Score, and the AUC were calculated. The minimum and maximum values, mean, and standard deviation of each metric were evaluated. The libraries used for algorithm training were Lazy Predict 0.2.12, Scikit-Learn 1.2.0 (Buitinck et al., 2011), and Tensorflow 2.10.1 (Abadi et al., 2016), executed in Python 3.9.

The first step involved running 100 simulations using the LazyClassifier function from the Lazy Predict 0.2.12 library, which automates the training of 27 different ML-based algorithms. These algorithms include: Stochastic Gradient Descent Classifier (SGD), Linear Support Vector Classification (Linear SVC), Logistic Regression, Linear Discriminant Analysis, Ridge Classifier with Cross Validation (CV), Ridge Classifier, AdaBoost Classifier, Nearest Centroid, Gaussian Naïve Bayes, Passive Aggressive Classifier, Nu Support Vector Classification (NuSVC), Light Gradient Boosting Machine Classifier (LGBM classifier), Quadratic Discriminant Analysis, Support Vector Classification (SVC), XGBoost Classifier, Bernoulli Naïve Bayes, KNeighbors Classifier, Random Forest Classifier, Bagging Classifier, Perceptron, Extra Trees Classifier, Extra Tree Classifier, Calibrated Classifier with CV, Label Spreading, Label Propagation, Decision Tree Classifier, Dummy Classifier.

The mean accuracy for each algorithm across each subset was recorded. This data-driven approach allowed for a comprehensive analysis to identify the most effective algorithms. Only the seven algorithms that demonstrated the highest mean accuracy were selected for further investigation. Similarly, the single subset that yielded the best accuracy across all algorithms was chosen for use in subsequent steps.

With the selected subset and 7 best-performing algorithms, 5,000 new simulations were conducted using the Scikit-Learn 1.2.0 library. Additionally, the same subset was employed to train 11 distinct dense neural networks architectures. Those networks differ in terms of the number of layers, the number of units per layer, and the dropout rate. By randomly excluding some units, dropout distributes learning across connections and helps reduce overfitting, improving model generalization and neural network performance when faced with unseen data (Salehin and Kang, 2023). The neural networks were built, trained and evaluated using the Tensorflow 2.10.1 library. To test the architecture with best performance, 100 simulations were conducted for each network. The hidden layers of the tested architectures were the following:

1. Single layer: 32 units with 20% dropout.
2. Two-layer: First layer with 32 units and 20% dropout, followed by a second layer with 16 units and 20% dropout.
3. Three-layer: Sequential layers with 32 units (20% dropout), 16 units (20% dropout), and 8 units (20% dropout).
4. Single layer: 16 units with 20% dropout.
5. Single layer: 8 units with 20% dropout.
6. Two-layer: First layer with 8 units and 20% dropout, followed by a second layer with 4 units and 20% dropout.
7. Four-layer: Layers configured with 64 units (20% dropout), 32 units (20% dropout), 16 units (10% dropout), and a final layer of 8 units without dropout.
8. Single layer: 8 units without dropout.
9. Two-layer: First layer with 16 units without dropout, followed by a second layer with 8 units without dropout.
10. Three-layer: Layers arranged with 16 units (no dropout), 8 units (no dropout), and a final layer of 4 units without dropout.
11. Two-layer: First layer with 8 units without dropout, followed by a second layer with 4 units without dropout.

All neural networks were trained using the same subset and therefore shared the same input layer configuration. Their hidden layers uniformly utilized ReLU activation, while their output layers consisted of a single unit with Sigmoid activation. The training process employed Adam optimization and Binary Cross-Entropy as the loss function over 20 epochs. The architecture that yielded the highest validation accuracy was selected for an additional 5,000 simulations.

## 3 Results

### 3.1 Cohort outcomes analysis

Out of the 537 study participants, 421 were confirmed to have a diagnosis of COVID-19. Among them, 133 experienced an outcome classified as severe (31.5%). It’s important to note that a single patient may have had multiple severe outcomes during the follow-up period, but they were treated the same way as the ones that had a single severe outcome.

### 3.2 Demographic analysis

The demographic profile of patient groups that progressed with severe and non-severe forms was analyzed, as described in Tables 1, 2. It can be observed that there is a predominance of female patients, of mixed ethnicity, and aged over 50 years old.

**Table 1:** Gender, race, and marital status distributions.

	Non severe (absolute risk)	Severe (absolute risk)	Total
**Gender**			
Male	128 (71.91%)	50 (28.09%)	178 (42.28%)
Female	160 (65.84%)	83 (34.16%)	243 (57.72%)
**Race**			
White	53 (68.83%)	24 (31.17%)	77 (18.29%)
Black	17 (73.91%)	6 (26.09%)	23 (5.46%)
Mixed	189 (66.55%)	95 (33.45%)	284 (67.46%)
Asian	5 (62.50%)	3 (37.50%)	8 (1.90%)
Indigenous	2 (66.67%)	1 (33.33%)	3 (0.71%)
Chose not to declare	19 (86.36%)	3 (13.64%)	22 (5.32%)
Blank/unfilled	3 (75.00%)	1 (25.00%)	4 (9.50%)
**Marital status**			
Single	72 (69.23%)	32 (30.77%)	104 (24.70%)
Married	150 (73.17%)	55 (26.83%)	205 (48.69%)
Divorced	26 (72.22%)	10 (27.78%)	36 (8.55%)
Widows	12 (57.14%)	9 (42.86%)	21 (4.99%)
Other	24 (52.17%)	22 (47.83%)	46 (10.93%)
Blank/unfilled	4 (44.44%)	5 (55.56%)	9 (2.14%)

**Table 2:** Age distribution.

	Non severe	Severe	Total
Mean	55.60	61.82	57.57
Median	55	62	57
Standard deviation	15.35	16.54	15.98
Blank/unfilled	1	0	1

### 3.3 Classifier results

During 100 simulations, the 7 subsets constructed in this study were used to train 27 ML algorithms using the Lazy Predict library. In each simulation, the training and test groups were randomly separated in an 80 to 20% ratio, respectively, and the accuracy of each simulation was computed. The average accuracy was then calculated for each classifier and each subset, resulting in the values shown in Table 3. It was possible to determine that the highest average accuracy was achieved with the Support Vector Classifier (SVC) for the 28-variable subset (subset F, without imputation), and the seven best classifiers for this subset were SVC, NuSVC, Random Forest Classifier, Ridge Classifier CV, Ridge Classifier, Extra Trees Classifier, and Linear Discriminant Analysis.

**Table 3:** Average accuracy after 100 simulations with each subset.

	Average accuracy per subset						
Classifier	A	B	C	D	E	F	G
XGBoost classifier	74.62%	77.25%	77.27%	77.32%	77.63%	77.20%	72.87%
Decision tree classifier	70.13%	70.13%	69.94%	70.19%	70.82%	71.44%	66.07%
Logistic regression	74.83%	77.51%	78.06%	77.63%	77.65%	77.72%	75.02%
AdaBoost classifier	73.77%	77.22%	75.87%	76.32%	76.30%	76.32%	72.72%
Bagging classifier	75.14%	77.33%	78.21%	77.54%	77.84%	77.08%	74.55%
Linear discriminant analysis	74.70%	77.90%	78.76%	78.16%	78.40%	78.38%	75.46%
Linear SVC	74.80%	77.63%	78.32%	77.68%	77.77%	77.34%	75.09%
SVC	76.30%	79.12%	79.90%	79.44%	80.12%	80.78%	77.27%
Passive aggressive classifier	67.48%	67.96%	70.24%	70.93%	69.42%	69.94%	67.05%
NuSVC	75.07%	78.84%	79.52%	79.09%	79.75%	80.14%	76.35%
Nearest centroid	68.04%	70.99%	71.95%	71.04%	70.58%	71.54%	69.78%
LGBM classifier	74.49%	77.33%	77.23%	78.26%	78.40%	77.46%	73.36%
Ridge classifier	74.68%	78.45%	78.84%	78.32%	78.51%	78.74%	75.52%
Ridge classifier CV	74.75%	78.42%	78.84%	78.35%	78.77%	79.04%	75.75%
Random forest classifier	76.20%	78.97%	80.18%	79.30%	80.33%	80.10%	76.74%
Gaussian NB	70.03%	71.70%	72.94%	71.00%	71.56%	71.78%	69.52%
Calibrated classifier CV	74.41%	77.88%	78.08%	76.67%	77.68%	76.90%	75.65%
Bernoulli NB	73.07%	74.36%	76.16%	75.68%	74.84%	76.10%	72.64%
Extra trees classifier	74.83%	77.72%	78.61%	78.49%	79.51%	79.56%	75.60%
Quadratic discriminant analysis	71.46%	72.51%	73.31%	72.04%	70.96%	68.82%	70.29%
Extra tree classifier	65.07%	69.19%	70.56%	68.60%	67.40%	67.74%	65.91%
Label spreading	67.75%	70.66%	74.06%	73.26%	73.35%	74.24%	67.69%
Label propagation	67.72%	70.70%	74.03%	73.26%	73.35%	74.24%	67.68%
KNeighbors classifier	73.00%	76.34%	77.65%	75.82%	75.70%	75.80%	73.19%
Perceptron	68.89%	70.13%	71.35%	70.82%	69.88%	70.80%	67.05%
SGD classifier	68.28%	71.54%	72.08%	72.18%	71.98%	71.38%	68.38%
Dummy classifier	68.68%	70.76%	71.16%	69.82%	71.30%	69.56%	68.32%

Subsets A-F address missing data through subject elimination, while subset G uses mean imputation.

Using the Scikit-Learn library, these seven classifiers were employed for 5,000 new simulations, once again with random redistributions between the training and test groups (Monte Carlo cross-validation). This library combines algorithm implementations based on previous works. In these simulations, only subset F was used (which includes 28 variables and has no imputation). Evaluation metrics were computed to calculate the mean and margin of error for a 95% Confidence Interval (CI 95).

In addition to the previously mentioned algorithms, 11 different configurations of dense neural networks were tested on this subset through 100 simulations with random resampling of the training and test groups. These simulations facilitated the identification of the most effective topology or neural network configuration. The classifier’s error can be described as a function of the chosen hyperparameters, such as the number of layers and units per layer. The objective is to minimize this error; however, due to computational constraints, it’s not feasible to exhaustively test all possible combinations. Consequently, 11 configurations with slight variations were selected, and their average accuracies were computed. The optimal configuration emerged as one with a single hidden layer containing 32 units and a 20% dropout rate. This configuration was subjected to the same training regimen as the seven highest-performing classical ML classifiers, undergoing a Monte Carlo Cross-Validation with 5,000 simulations.

Table 4 describes the results of each algorithm after 5,000 trainings. Very similar values were observed among different algorithms, with a very small margin of error around the average accuracy (CI 95). The high specificity, combined with a significant positive predictive value of the SVC, indicates a high success rate when the algorithm classifies patients as severe. On the other hand, the Ridge Classifier, Ridge CV, and Linear Discriminant Analysis algorithms maintain accuracy while achieving better results in identifying patients who will not progress to the severe form. The neural network, however, exhibited low sensitivity and lower accuracy compared to the other methods. An important metric for classifier evaluation, the AUC, indicates that the tested classifiers had less satisfactory performance in distinguishing between classes. An exception was the Random Forest, with an average AUC of 0.91 and an average accuracy of 80%.

**Table 4:** Average performance metrics from 5,000 simulations using subset F (with 28 variables, no imputation).

Classifier	Accuracy	F1-score	Positive predicted value	Negative predicted value	Sensitivity	Specificity	AUC
SVC	0.79 ± 0.0014	0.54 ± 0.0031	0.87 ± 0.0036	0.78 ± 0.0016	0.40 ± 0.0031	0.97 ± 0.0008	0.79 ± 0.0020
NuSVC	0.79 ± 0.0015	0.56 ± 0.0032	0.83 ± 0.0037	0.79 ± 0.0018	0.44 ± 0.0036	0.96 ± 0.0010	0.78 ± 0.0022
Random forest classifier	0.80 ± 0.0014	0.58 ± 0.0031	0.81 ± 0.0039	0.79 ± 0.0017	0.46 ± 0.0034	0.95 ± 0.0011	0.91 ± 0.0014
Extra trees classifier	0.79 ± 0.0014	0.57 ± 0.0031	0.77 ± 0.0039	0.79 ± 0.0017	0.47 ± 0.0035	0.94 ± 0.0012	0.78 ± 0.0019
Ridge classifier CV	0.80 ± 0.0015	0.63 ± 0.0028	0.75 ± 0.0037	0.82 ± 0.0017	0.56 ± 0.0034	0.91 ± 0.0014	0.77 ± 0.0020
Ridge classifier	0.78 ± 0.0015	0.62 ± 0.0028	0.69 ± 0.0038	0.82 ± 0.0017	0.57 ± 0.0033	0.88 ± 0.0016	0.77 ± 0.0020
Linear discriminant analysis	0.78 ± 0.0015	0.61 ± 0.0027	0.67 ± 0.0036	0.82 ± 0.0017	0.58 ± 0.0034	0.87 ± 0.0017	0.77 ± 0.0020
Artificial neural network	0.76 ± 0.0016	0.45 ± 0.0034	0.81 ± 0.0050	0.76 ± 0.0018	0.33 ± 0.0033	0.96 ± 0.0012	0.77 ± 0.0018

In a context of multiple hospitalized patients and resource scarcity, such as during the COVID-19 pandemic, the achieved predictive values would be extremely useful. The 87% positive predictive value attained by the SVC, coupled with a small number of false positives, allows for better resource allocation and interventions, such as surveillance or transfer to another healthcare unit. It is essential to note that only admission data were used for this analysis, and clinical follow-up during hospitalization, along with additional tests, could lead to patient reclassification and enhance system performance. Also, since the data originated from a prospective cohort, in newer studies the classifiers could be implemented during the cohort, guiding data collection toward most important variables.

Classifiers for COVID-19 severity prediction have been published with accuracies ranging from 74.4 to 95.20% and AUC values between 0.66 and 0.997 (Shakibfar et al., 2023; Wang et al., 2021). However, a significant portion of this research relied on data from retrospective and/or single-center studies, limiting the generalizability of the findings (Wang et al., 2021). In our work, we conducted a prospective multicenter study aimed at ensuring that this methodology can be applied in real-time during new episodes of public health crises.

The availability of data during public health crises poses significant challenges. Resource scarcity in various healthcare centers limits not only the number of clinical variables that can be collected but also the ability to leverage predictive models that require these variables. Previous studies often utilized data collected from healthcare services with greater resource availability, resulting in a higher number of tests performed and fewer missing data. This situation, however, does not reflect the reality of many healthcare centers in underdeveloped or developing countries. By utilizing only the most readily available variables in healthcare settings of different complexity levels, we enable the application of the predictor even in adverse situations.

Ramírez-Del Real et al. (2022) also achieved satisfactory results by using demographic, clinical, and laboratory data to predict COVID-19 mortality. Data were collected from healthy individuals who were subsequently monitored to evaluate outcomes if they contracted COVID-19. They achieved an accuracy of 90.41%, with positive and negative predictive values of 94.28 and 87.36%, respectively. This study suggests the possibility of identifying individuals more vulnerable to unfavorable outcomes even before contracting the disease.

### 3.4 Contributing features

After training, we selected the two models that demonstrated the best performance, Random Forest and SVC, to extract the features that contributed most significantly to the classification process. For the Random Forest model, we averaged the “feature importance” values for each variable across the 5,000 simulations. In the case of the SVC model, which was implemented using the Radial Basis Function kernel, we employed the Permutation Feature Importance technique, as implemented in Scikit-Learn. The Permutation Importance values for each variable were computed for each simulation, and we used the average values to identify the most significant features. To conclude our analysis, we performed logistic regression on the same subset used to train the models and calculated the *p*-values associated with each variable. These values are presented in Table 5.

**Table 5:** Feature importance and *p*-values for classification models.

Feature	Importance RF	Importance SVC	*p*-values (logistic regression)
General condition of the patient upon admission	0.1158	0.0298	< 0.05
Age (years)	0.0696	0.0040	0.1607
Number of leukocytes (number/mm^3^)	0.0324	0.0009	0.7348
Number of lymphocytes (number/mm^3^)	0.0185	0.0015	0.3840
Number of platelets (number/mm^3^)	0.0388	0.0037	< 0.05
Urea (mg/dL)	0.0478	0.0108	< 0.05
Systolic blood pressure upon admission (mmHg)	0.0435	−0.0022	0.0565
Diastolic blood pressure upon admission (mmHg)	0.0396	0.0013	0.4980
Heart rate upon admission (bpm/min)	0.0302	0.0031	0.4154
Respiratory Rate UPON admission (breaths/min)	0.0559	0.0027	0.7092
Oxygen saturation upon admission (%)	0.0621	0.0144	< 0.05
No pre-existing conditions	0.0203	0.0002	0.4987
Hypertension	0.0247	−0.0006	0.2366
Diabetes mellitus	0.0306	0.0032	0.1365
Obesity	0.0086	−0.0008	0.6146
Kidney failure	0.0045	−0.0001	0.6052
Fever	0.0221	0.0017	0.1207
Dry cough	0.0218	−0.0009	0.0562
Headache	0.0201	0.0033	0.0757
Weakness	0.0162	0.0004	0.3476
Anosmia	0.0103	0.0002	0.5427
Gender	0.0215	−0.0009	0.4630
Signs of upper respiratory tract infection (URTI)	0.0239	0.0034	< 0.05
Dyspnea	0.1334	0.0656	< 0.05
Signs of pulmonary involvement	0.0214	−0.0006	0.7351
Constitutional symptoms	0.0226	0.0002	0.7766
Previous pulmonary diseases	0.0070	−0.0007	0.1377
Hemoglobin (g/dL)	0.0368	−0.0005	0.7695

The table displays features, their importance in Random Forest, importance in SVC, and *p*-values from logistic regression.

The most statistically significant variables for predicting severe outcomes, with a p-value less than 0.05, included dyspnea, the patient’s general condition upon hospital admission, peripheral oxygen saturation, urea levels, platelet count, and signs of upper respiratory tract infection. In the Random Forest model, the most important features were dyspnea, general condition of the patient upon admission, age, oxygen saturation upon admission, respiratory rate upon admission, and urea. For the SVC model, the significant features included dyspnea, general condition of the patient upon admission, oxygen saturation upon admission, urea, age, and platelet count.

These results share some similarities with findings from previous studies, where age and platelet count were identified as significant predictors of prognosis in severely hospitalized patients (Martono and Mulyanti, 2023; Fang et al., 2020; Kamel et al., 2023). Additionally, other important features, such as different hematological variables, D-dimer and C-reactive protein, could further enhance the predictive power of the system. However, these tests were unavailable for a significant portion of the studied population.

## 4 Discussion

An extensive data preprocessing process was carried out to identify as many filling errors or duplicates as possible. Additionally, the variable selection process involved not only searching for better classifier performance but also minimizing the loss of individuals after data cleaning. Constructing seven different subsets, each encompassing varying numbers of variables and consequently individuals, allowed us to observe the effect on classifier outcomes.

When assessing the cost-effectiveness of using the developed classifiers, it is crucial to consider that laboratory tests and clinical evaluations are indispensable for proper inpatient monitoring. Therefore, the use of the classifier would not incur additional expenses beyond an informatics system capable of generating results. The utilization of these classifiers does not require additional tests beyond those essential for usual medical follow-up.

During preprocessing, a test was conducted to assess the impact of imputation for filling missing data. The method used was to fill in missing data with the means calculated for all individuals. In other words, those who did not have their hemoglobin measured received the average hemoglobin value from all individuals who had the test collected. Imputation enables certain machine learning algorithms that do not accept incomplete input information to be applied to all study participants. However, it was observed that mean imputation reduced classifier accuracy. It is understood that clinical data variables are not independent of each other, and therefore, alternative data imputation methods could yield better results. Through multivariate analysis, the tests collected for a specific individual could be used to estimate the missing values. For instance, hemoglobin, respiratory rate, and clinical signs of dyspnea could be utilized to estimate peripheral oxygen saturation (SpO2) for patients who could not undergo oximetry. Further dedicated studies are recommended to identify optimal clinical data imputation methods for classifier development.

It has been observed that reducing input variables, despite decreasing the loss of individuals (due to incompleteness or missing data) and the complexity of classifier systems, compromises the accuracy of classification systems. Throughout this work, experimentation was necessary to find the best solution for this problem. Tests with different subsets helped strike the right balance between individual losses and the amount of clinical data used. A high loss of individuals would hinder classifier learning, as well as the reliability and generalizability of results. The reliance on a large amount of clinical data, coupled with the inability of most algorithms to handle incompleteness, may indicate inferior performance compared to a trained professional. This reinforces the idea that AI should be viewed as a tool rather than a replacement for human expertise.

The high number of sparse variables collected during the cohort study draws some attention. Several variables were positive in less than 1% of study participants, such as the presence of arrhythmia or prior valvular disease. This characteristic poses a particular challenge when developing a classifier. The more variables used as input, the more complex the classifier becomes. Even if we imagine a simple questionnaire for diagnosing a specific disease, the more questions required for the diagnosis, the more challenging its implementation becomes, during real healthcare situations. Also, complex classifiers are more prone to overfitting, which reduces their ability to generalize to new data and patients (Pansambal and Nandgaokar, 2023; Salehin and Kang, 2023). Simultaneously, some clinical data, despite being rare, can serve as excellent markers for unfavorable outcomes in certain pathologies. Excluding these rare data points from the analysis could compromise the quality of the final analysis. Considering this, clinical knowledge of signs and symptoms, as potential severity predictors (Ou et al., 2020; Martono and Mulyanti, 2023; Fang et al., 2020), was considered during variable selection, along with combining different variables with the same clinical significance (such as a variable representing the presence of any prior lung disease).

Regarding AI-based classifiers, it is essential to consider that a significant class imbalance can hinder the development of classification systems (Isgut et al., 2023). In this study, approximately 69% of participants did not experience severe complications. Consequently, a classifier that labeled all individuals as “non-severe” would achieve a considerable accuracy rate. However, based on the results obtained, it is possible to affirm that satisfactory accuracy models were developed, particularly when classifying an individual as belonging to the “severe” group. This is evident from the high positive predictive value and specificity.

Specificity and sensitivity are crucial for determining the quality of a medical screening test. When both are high, it indicates a low rate of classification errors. Despite the low sensitivity of the developed classifiers, the high specificity suggests a small number of false positives. Few false positives are generally preferable, even at the expense of reduced sensitivity, especially when a positive test result could lead to unnecessary and dangerous interventions (Herman, 2006; Trevethan, 2017).

Using data collected from a multicenter study, it would not be inaccurate to assume that the developed system would have greater generalization capacity. This is because the study involved healthcare units with varying capabilities, technological density, and resource access. Consequently, the system could be used in both less-structured hospitals and more comprehensive units. It is also worth emphasizing that the data were collected from a prospective cohort, ensuring greater reliability regarding data quality, despite adding technical limitations, such as collecting all variables from all participants. No other studies were found that solely utilized clinically and demographically collected information in a prospective manner. Furthermore, due to its adaptation to real-world constraints and its inclusion of individuals from diverse socioeconomic backgrounds and demographic profiles, the developed system has greater practical usability and can span different levels of healthcare units.

In recent years, several studies have been published addressing the prediction of unfavorable outcomes in COVID-19 using AI systems (Kim, 2022; Yu et al., 2021; Shakibfar et al., 2023; Wang et al., 2021; Ramírez-Del Real et al., 2022; Kamel et al., 2023). One of the challenges encountered in the literature on ML and AI for disease diagnosis or prognosis evaluation is the choice of performance metrics. Few studies have reported positive and negative predictive values, and some have not even published sensitivity and specificity values (Wang et al., 2021). While a new clinical study is necessary to precisely determine these metrics using data from new, previously unseen patients, calculating these probabilities was considered essential to assess the viability of the classifiers. It is considered that AI-based algorithms for diagnosis should be evaluated similarly to new laboratory and imaging tests.

Other studies have also demonstrated satisfactory results by developing predictors that incorporate imaging exams, such as Computed Tomography (CT), alongside clinical data to classify COVID-19 severity (Yu et al., 2021). The cohort described in this study also collected bedside chest radiographies, which could be combined with clinical data to enhance results in future work. However, by utilizing only a few variables resulting from simple tests, the prediction system could be used even in healthcare units with low technological density. It is estimated that in 2020, only approximately 15% of Brazilian municipalities had access to Computed Tomography (Pereira and Tomógrafos, 2020).

This work considers healthcare systems with resource scarcity and the reality of developing and underdeveloped countries. Nevertheless, this methodology can be adjusted to meet the needs of different countries, such as including collaboration between various governments and organizations in data collection, as well as steering data collection toward more widely available tests.

It is important to emphasize that the objective of these AI-based systems is not to replace healthcare professionals but to assist in the triaging process during high-demand situations. By providing timely and accurate support, these systems can help alleviate the workload of medical staff, allowing them to focus on critical decision-making and patient care. The collaboration between AI and healthcare providers can enhance overall efficiency and effectiveness in delivering quality care.

This classifier was designed to operate specifically in moderate and severe cases of COVID-19 that required hospitalization. Mild cases of COVID-19 typically do not require the tests we used and often do not seek healthcare services. While this classification could potentially be applied to other respiratory diseases, further validation with new data will be necessary to confirm its effectiveness in those contexts. Ongoing research and data collection will be crucial to refining and adapting the classifier for broader applications in managing other diseases.

## 5 Conclusion and future work

It is understood that vaccination and the emergence of new SARS-CoV-2 variants alter the disease’s behavior and the number of severe complications. A reduction in severe COVID-19 cases has been observed in vaccinated individuals, while the transmission has increased with the emergence of new variants (Martono and Mulyanti, 2023). Consequently, collecting new data and updating classifiers becomes necessary.

Given its multidisciplinary nature, it is essential to emphasize the importance of going beyond preprocessing and classification algorithms by incorporating medical expertise. The fusion of medical and computational perspectives allows for a more comprehensive analysis of the feasibility, applicability, and validity of the classification system.

This work leaves a few gaps that must be addressed in future research before implementing this type of classification system in healthcare. Firstly, although mean imputation was utilized to manage missing data, more sophisticated techniques, such as multivariate analysis, may yield improved results. Secondly, the prevalence of numerous sparse variables – those that are positive in only a small proportion of patients – should be considered in future data collection efforts. By adjusting the form and method of data collection to focus on gathering more general information rather than numerous specific variables, we can reduce the complexity of the cohort study while addressing the challenges AI algorithms encounter with sparse data. Additionally, the study revealed challenges related to class imbalance, with a greater number of patients classified as “non-severe,” which may hinder the classifiers’ ability to accurately identify severe cases. This suggests the need for further exploration of sampling techniques or specialized algorithms. Moreover, it is essential to investigate the potential benefits of incorporating imaging data, such as chest X-rays or computed tomography scans, as well as the impact of vaccination and emerging SARS-CoV-2 variants on prediction accuracy. Finally, external validation using diverse datasets and assessing the performance of the developed system in prospective studies is crucial before it can be effectively implemented in clinical practice.

This study validated the methodology for developing predictors of morbidity and mortality, from data collection to training machine learning algorithms. This methodology can be applied to other diseases, especially those where outcomes depend on the interaction of multiple variables.

## Data Availability

The original contributions presented in the study are included in the article/supplementary material, further inquiries can be directed to the corresponding author.
